# 108 Implementation of a Screening Instrument Assessing Risks and Social Determinants of Health: Feasibility, Findings, Implications

**DOI:** 10.1093/jbcr/irae036.107

**Published:** 2024-04-17

**Authors:** Ashley B Hink, Steven A Kahn, Rohit Mittal, Haley E Konsek, Anjali Mohan, Yulia Gavrilova

**Affiliations:** Medical University of South Carolina, Charleston, South Carolina; MUSC, Charleston, SC; MUSC/SJCH, Charleston, SC; South Carolina Burn Center / Medical University of South Carolina, Charleston, South Carolina; Medical University of South Carolina, Charleston, South Carolina; MUSC, Charleston, SC; MUSC/SJCH, Charleston, SC; South Carolina Burn Center / Medical University of South Carolina, Charleston, South Carolina; Medical University of South Carolina, Charleston, South Carolina; MUSC, Charleston, SC; MUSC/SJCH, Charleston, SC; South Carolina Burn Center / Medical University of South Carolina, Charleston, South Carolina; Medical University of South Carolina, Charleston, South Carolina; MUSC, Charleston, SC; MUSC/SJCH, Charleston, SC; South Carolina Burn Center / Medical University of South Carolina, Charleston, South Carolina; Medical University of South Carolina, Charleston, South Carolina; MUSC, Charleston, SC; MUSC/SJCH, Charleston, SC; South Carolina Burn Center / Medical University of South Carolina, Charleston, South Carolina; Medical University of South Carolina, Charleston, South Carolina; MUSC, Charleston, SC; MUSC/SJCH, Charleston, SC; South Carolina Burn Center / Medical University of South Carolina, Charleston, South Carolina

## Abstract

**Introduction:**

Mental illness and substance abuse (SA) are risk factors for burns and associated with poor outcomes. Low health literacy, adverse social determinants of health (SDoH), community level disparity, and intimate partner violence (IPV) are associated with poor outcomes, injury recidivism and readmissions in other patient populations. There are currently no formalized strategies for assessment of such factors in burn centers. This study examines the implementation of a standardized screening instrument for risks and SDoH, their prevalence, supportive services provided, and factors associated with unplanned emergency department (ED) visits and readmissions.

**Methods:**

A screening assessment for health literacy, 4 specific SDoH, depression, suicidality, SA and IPV was integrated into the care of burn patients. Patient addresses were linked to the Area Deprivation Index (ADI, range 1-10). Registry and medical record data for adult patients treated March 2022 – March 2023 were retrospectively reviewed. Data include clinical characteristics, demographics, screening implementation and results, supportive services, unplanned ED visits and readmissions. Descriptive statistics were used and between group differences were assessed using student’s T-tests and Chi-Square analyses with statistical significance defined as p< 0.05.

**Results:**

305 patients were included for analysis. The mean age was 48.8, TBSA was 6.7%, LOS was 10.3 days, and ADI was 5. 215 of patients (70.5%) received screening by members of the clinical team. Of those screened, 22.4% had low or inadequate health literacy, 24% were positive for SA, 9% for depression, 10.7% for suicidality, 10.7% for IPV, and 22% had at least one adverse SDoH. Patients that were screened received more supportive in-hospital services (i.e. SA peer counselor, IPV social worker, psychologist). 58 (19%) and 42 (13.8%) of patients had unplanned ED visits and readmissions, respectively. 34.5% of ED visits and 66.7% of readmissions were related to the index injury. ADI was not associated with unanticipated ED visits or admissions. Patients screening positive for depression, SA, suicidality, housing instability, food insecurity, and transportation barriers were significantly more likely to have an unanticipated all-cause ED visit following discharge, but not readmission.

**Conclusions:**

A screening tool was administered to 70.5% of patients to identify risks and adverse SDoH that ranged in prevalence from 9-24%, and increased in-hospital supportive services. Screening results were not associated with repeat admissions, but some were associated with increased risk of all-cause ED visits after discharge.

**Applicability of Research to Practice:**

This study demonstrates feasibility of a universal screening tool in the burn population that identifies risks and SDoH, and can help clinical teams mobilize hospital resources to reduce poor outcomes. Supportive services after discharge may reduce ED visits related to other causes.

